# *MYC, FBXW7* and *TP53* copy number variation and expression in Gastric Cancer

**DOI:** 10.1186/1471-230X-13-141

**Published:** 2013-09-23

**Authors:** Danielle Queiroz Calcagno, Vanessa Morais Freitas, Mariana Ferreira Leal, Carolina Rosal Teixeira de Souza, Samia Demachki, Raquel Montenegro, Paulo Pimentel Assumpção, André Salim Khayat, Marília de Arruda Cardoso Smith, Andrea Kely Campos Ribeiro dos Santos, Rommel Rodriguez Burbano

**Affiliations:** 1 Laboratório de Citogenética Humana, Instituto de Ciências Biológicas, Universidade Federal do Pará, Belém, PA, Brasil; 2 Disciplina de Genética, Departamento de Morfologia e Genética, Escola Paulista de Medicina, Universidade Federal de São Paulo, Rua Botucatu 740, CEP 04023-900, São Paulo, SP, Brazil; 3 Departamento de Biologia Celular e do Desenvolvimento, Instituto de Ciências Biomédicas, Universidade de São Paulo, São Paulo, SP, Brasil; 4 Laboratorio de Imunoistoquímica, Serviço de Anatomia Patológica, Faculdade de Medicina, Hospital Universitário João de Barros Barreto, Universidade Federal do Pará, Belém, PA, Brasil; 5 Serviço de Cirurgia Hospital Universitário João de Barros Barreto, Universidade Federal do Pará, Belém, PA, Brasil; 6 Laboratório de Genética Humana, Instituto de Ciências Biológicas, Universidade Federal do Pará, Belém, PA, Brasil

**Keywords:** Gastric cancer, MYC, FBXW7, TP53

## Abstract

**Background:**

MYC deregulation is a common event in gastric carcinogenesis, usually as a consequence of gene amplification, chromosomal translocations, or posttranslational mechanisms. *FBXW7* is a p53-controlled tumor-suppressor that plays a role in the regulation of cell cycle exit and reentry via MYC degradation.

**Methods:**

We evaluated *MYC*, *FBXW7*, and *TP53* copy number, mRNA levels, and protein expression in gastric cancer and paired non-neoplastic specimens from 33 patients and also in gastric adenocarcinoma cell lines. We also determined the invasion potential of the gastric cancer cell lines.

**Results:**

*MYC* amplification was observed in 51.5% of gastric tumor samples. Deletion of one copy of *FBXW7* and *TP53* was observed in 45.5% and 21.2% of gastric tumors, respectively. *MYC* mRNA expression was significantly higher in tumors than in non-neoplastic samples. *FBXW7* and *TP53* mRNA expression was markedly lower in tumors than in paired non-neoplastic specimens. Moreover, deregulated *MYC* and *FBXW7* mRNA expression was associated with the presence of lymph node metastasis and tumor stage III-IV. Additionally, MYC immunostaining was more frequently observed in intestinal-type than diffuse-type gastric cancers and was associated with *MYC* mRNA expression. In vitro studies showed that increased MYC and reduced FBXW7 expression is associated with a more invasive phenotype in gastric cancer cell lines. This result encouraged us to investigate the activity of the gelatinases MMP-2 and MMP-9 in both cell lines. Both gelatinases are synthesized predominantly by stromal cells rather than cancer cells, and it has been proposed that both contribute to cancer progression. We observed a significant increase in MMP-9 activity in ACP02 compared with ACP03 cells. These results confirmed that ACP02 cells have greater invasion capability than ACP03 cells.

**Conclusion:**

In conclusion, FBXW7 and MYC mRNA may play a role in aggressive biologic behavior of gastric cancer cells and may be a useful indicator of poor prognosis. Furthermore, MYC is a candidate target for new therapies against gastric cancer.

## Background

Gastric cancer (GC) is the fourth most common cancer and the second leading cause of cancer death worldwide [1]. GC is considered a major public health concern, especially in developing countries, including Brazil [2].

A fundamental aspect of carcinogenesis is uncontrolled cell proliferation resulting from the accumulation of changes that promote the expression or repression of cell cycle-control genes [3]. *MYC* is a transcriptional factor involved in cell cycle regulation and cell growth arrest that is commonly deregulated in cancers and has been described as a key element of gastric carcinogenesis [4,5]. Several different types of posttranslational modifications of MYC have been described, including phosphorylation, acetylation, and ubiquitination [6]. The ubiquitin-proteasome system is the major protein degradation regulatory pathway involved in cell differentiation and growth control [7]. *FBXW7* encodes an F-box protein subunit of the Skp1/Cul1/F-box complex (SCF) ubiquitin ligase complex. SCF^FBXW7^ induces degradation of the products of positive cell cycle regulator genes, such as *cyclin E*, *MYC*, *NOTCH*, and *JUN*, through phosphorylation-dependent ubiquitination [8]. Among SCF^FBXW7^ substrates, MYC is of particular importance in cell cycle exit because it is thought to play a role in determining whether mammalian cells divide or not [9].

Deregulated *FBXW7* expression is a major cause of carcinogenesis [10-12]. Loss of *FBXW7* expression can lead to MYC overexpression and has been associated with poor prognosis in GC patients [13]. However, MYC activation by *FBXW7* loss triggers activation of p53, which plays a key role in the regulation of cellular responses to DNA damage and abnormal expression of oncogenes. Induction of cell cycle arrest by p53 allows for DNA repair or apoptosis induction [14]. Thus, concomitant loss of *FBXW7* and *TP53* is necessary to induce genetic instability and tumorigenesis [11].

In the present study, we investigated *MYC*, *FBXW7*, and *TP53* gene copy number variation and mRNA and protein expression in GC samples and gastric adenocarcinoma cell lines. Possible associations between our findings and the clinicopathological features and/or invasion and migration capability of the cell lines were also evaluated.

## Methods

### Clinical samples

Samples were obtained from 33 GC patients who underwent surgical treatment at the João de Barros Barreto University Hospital in Pará State, Brazil. Dissected tumor and paired non-neoplastic tissue specimens were immediately cut from the stomach and frozen in liquid nitrogen until RNA extraction.

The clinicopathological features of the patient samples are shown in Table 1. GC samples were classified according to Lauren [15]. All GC samples showed the presence of *Helicobacter pylori*, and the cagA virulence factor was determined by PCR analysis of *ureA* and *cagA* as described by Clayton *et al*. [16] and Covacci *et al.*[17], respectively. All patients had negative histories of exposure to either chemotherapy or radiotherapy before surgery, and there were no other co-occurrences of diagnosed cancers. Informed consent with approval of the ethics committee of the Federal University of Pará was obtained.

**Table 1:** ***MYC*, *FBXW7 *and *TP53 *gene copy number variation, MYC and p53 protein expression and clinicopathological features of 33 GC patients**

	**CNV MYC**			**CNV FBXW7**			**CNV TP53**			**IHC MYC**			**IHC p53**		
	**2 copies (n = 16)**	**≥ 3 copies (n = 17)**	***p- value***	**2 copies (n = 18)**	**1 copy (n = 15)**	***p- value***	**2 copies (n = 25)**	**1 copy (n = 7)**	***p- value***	**P (n = 19)**	**N (n = 14)**	***p- value***	**P (n = 6)**	**N (n = 25)**	***p- value***
**Age (y) (mean ± SD)**															
>50 (65.3 ± 9.1)	7	12	0.166	12	7	0.304	15	3	0.393	10	4	0.310	5	9	0.094
≤50 (42.1 ± 8.2)	9	5		6	8		10	4		10	9		2	17	
**Gender**															
Male	8	7	0.437	7	8	1.000	13	1	0.104	12	3	0.072	2	13	0.413
Female	8	10		8	10		12	6		8	10		5	13	
**Histopathology**															
Intestinal	12	10	0.465	12	10	1.000	16	6	0.387	17	5	0.009*	6	16	0.378
Diffuse	4	7		6	5		9	1		3	8		1	10	
**Depth of tumor invasion**															
T1	3	3	1.000	5	1	0.186	5	1	1.000	2	4	0.182	1	5	1.000
T2-T4	13	14		13	14		20	6		18	9		6	21	
**Lymph node metastasis**															
Absent	5	8	0.481	8	5	0.722	10	3	1.000	7	6	0.717	2	11	0.676
Present	11	9		10	10		15	4		13	7		5	15	
**Stage**															
I-II	8	10	0.732	12	6	0.170	14	3	0.678	12	6	0.493	3	15	0.674
III-IV	8	7		6	9		11	4		8	7		4	11	
**MYC IHC**															
Negative	5	8	0.481	7	6	1.000	11	2	0.671						
Positive	11	9		11	9		14	5							
**p53 IHC**															
Negative	14	12	0.398	14	12	1.000	21	4	0.157						
Positive	2	5		4	3		4	3							

*p < 0.05; P: positive; N: negative.

### Cells lines

Gastric adenocarcinoma cell lines ACP02 and ACP03 [18] were cultured in complete RPMI medium (Invitrogen Corp., Carlsbad, CA, USA) supplemented with 10% fetal bovine serum (FBS), 1% penicillin/streptomycin, and 1% kanamycin.

### Copy number variation (CNV)

DNA was extracted using a DNAQiamp mini kit (Qiagen, Hilden, Germany) according to the manufacturer’s instructions. Duplex quantitative real-time PCR (real-time qPCR) was performed using the FAM/MGB-labeled TaqMan probes for *MYC* (Hs01764918_cn), *FBXW7* (Hs01362464_cn), or *TP53* (Hs06423639_cn), and VIC/TAMRA-labeled TaqMan CNV *RNAse P* (#4403326) was used for the internal control. All real-time qPCR reactions were performed in quadruplicate with gDNA according to the manufacturer’s protocol using a 7500 Fast Real-Time PCR system (Life Technologies, Foster City, CA, USA). The copy number of each sample was estimated by CNV analysis using Copy Caller Software V1.0 (Life Technologies, Foster City, CA, USA). Known Human Genomic DNA (Promega, Madison, USA) was used for calibration.

### Quantitative real-time reverse transcriptase PCR

Total RNA was extracted with TRI Reagent^®^ Solution (Life Technologies, Carlbad, CA, USA) following the manufacturer’s instructions. RNA concentration and quality were determined using a NanoDrop spectrophotometer (Thermo Scientific, Wilmington, DE, USA) and 1% agarose gels. Complementary DNA (cDNA) was synthesized using a High-Capacity cDNA Archive kit according to the manufacturer’s recommendations (Life Technologies, Foster City, CA, USA). Real-time qPCR primers and TaqMan probes targeting *MYC* (Hs00153408_m1), *FBXW7* (Hs00217794_m1), and *TP53* (Hs01034249_m1) were purchased as Assays-on-Demand Products for Gene Expression ((Life Technologies, Foster City, CA, USA). Real time qPCR was performed using an ABI Prism 7500 system (Life Technologies, Foster City, CA, USA) according to the manufacturer’s instructions. *GAPDH* (NM_002046.3; Life Technology, USA) was selected as an internal control for monitoring RNA input and reverse transcription efficiency. All real-time qPCR reactions for target genes and internal controls were performed in triplicate on the same plate. The relative quantification (RQ) of gene expression was calculated using the ΔΔCt method [19], in which the non-neoplastic sample was designated as a calibrator for each paired tumor sample.

### Immunohistochemistry

Immunohistochemical analyses for MYC and p53 were performed on formalin-fixed, paraffin-embedded surgical sections. Serial 3-μm sections were used. Heat-induced antigen retrieval was employed (microprocessor-controlled pressure Pascal^®^ DakoCytomation, Carpinteria, CA, USA). A universal peroxidase-conjugated secondary antibody kit (LSAB System, DakoCytomation, Carpinteria, CA, USA) was used for detection with diaminobenzidine (DAB) as the chromogen. The following primary antibodies were used: mouse monoclonal antibodies directed against MYC (dilution 1:150; sc-40, Santa Cruz Biotechnology, Santa Cruz, CA, USA and clone 9E10, Zymed^®^, San Francisco, CA, USA), FBXW7 (dilution 1:50, Abnova Corp., Taipei City, Taiwan), and p53 (dilution 1:50; DakoCytomation, Carpinteria, CA, USA). Positive protein expression was defined as clear nuclear staining in more than 10% of the cells.

### Migration and invasion assay

Migration and invasion assays were carried out in a modified Boyden chamber with filter inserts (8-μm pores) for 12-well plates (BD Biosciences, San Jose, CA, USA). To assess invasion, filters were coated with 10 μl of Matrigel (10–13 mg/ml) (BD Biosciences, San Jose, CA, USA) while on ice. Cells (2 × 10^5^) were plated into the upper chamber in 1 ml of RPMI without FBS. The lower chamber was filled with 1.5 ml of RPMI with FBS. After 48 h in culture, cells were fixed with 4% paraformaldehyde and post-fixed with 0.2% crystal violet in 20% methanol. Cells on the upper side of the filter, including those in the Matrigel, were removed with a cotton swab. Invading cells (on the lower side of the filter) were photographed and counted. Experiments were performed in triplicate.

### Immunofluorescence

Cells grown on glass coverslips were fixed with 1% paraformaldehyde in phosphate-buffered saline (PBS) for 10 min, then permeabilized with 0.5% Triton X-100 (Sigma-Aldrich, St. Louis, MO, USA) in PBS for 15 min and blocked with 1% bovine serum albumin (BSA) in PBS. The cells were stained with mouse antibodies against MYC (diluted 1:50; Zymed^®^, USA), p53 (diluted 1:50; DakoCytomation, Carpinteria, CA, USA), and FBXW7 (diluted 1:50; Abnova Corp., Taipei City, Taiwan). Primary antibodies were revealed using an anti-mouse Alexa-568-conjugated secondary antibody (Invitrogen). All incubations were carried out for 60 min at room temperature. Nuclei were stained with DAPI in Prolong anti-fade mounting medium (Invitrogen). Negative control samples were processed as described above except that primary antibodies were omitted and replaced with PBS alone.

### Western blotting

Protein extraction from cells was performed according to standard procedures. Briefly, total protein was extracted from ACP02 and ACP03 cells using 50 mM Tris–HCl buffer containing 100 mmol/L NaCl, 50 mM NaF, 1 mM NaVO_4_, 0.5% NP-40, and complete protease inhibitor cocktail (Roche, Germany). Protein concentration was estimated using a Bradford assay (Sigma-Aldrich). About 30 μg of total protein extract was loaded onto a 12% sodium dodecyl sulfate-polyacrylamide gel electrophoresis (SDS-PAGE) gel and electrophoresed. Resolved proteins were then transferred from the gel onto a nitrocellulose membrane. The membrane was blocked with 5% nonfat milk in Tris-buffered saline containing 5% Tween (Sigma-Aldrich, Sant Louis, MO, USA) and then incubated with mouse monoclonal anti-MYC (Santa Cruz Biotechnology), anti-FBXW7 (Abnova, Taipei City, Taiwan), anti-p53 (DakoCytomation, Carpinteria, CA, USA), and anti-β-actin (Sigma-Aldrich, Sant Louis, MO, USA) antibodies diluted 1:200, 1:100, 1:100, and 1:2,000, respectively. Subsequently, membranes were incubated with a 1:5,000 dilution of horseradish peroxidase (HRP)-conjugated sheep anti-mouse antibody (Amersham Biosciences, Piscataway, NJ, USA) for 1 h at room temperature. Proteins were visualized by enhanced chemiluminescence.

### Zymography

ACP02 and ACP03 cells (5 × 10^4^ of each) were plated and allowed to adhere and spread for at least 8 h. Adherent cells were washed three times with PBS, and the culture medium was replaced with serum-free medium for 24 h. The activity of MMP2 and MMP9 in the conditioned medium was assessed by zymography. Conditioned medium was collected, concentrated (Microcon 30 K, Merck Millipore, Darmstadt, Germany) and resuspended in SDS-PAGE sample buffer (without β-mercaptoethanol). The remaining cells were lysed and the protein concentration was estimated using a BCA assay (Thermo Scientific Pierce, Rockford, IL, USA). A total of 1 μg of protein from each conditioned medium was separated on 10% polyacrylamide gels containing 0.2% gelatin. After electrophoresis, the gels were washed in 2.5% Triton X-100 for 30 min, then equilibrated in 10 mM Tris (pH 8.0) and incubated at 37°C for 16–24 h in a development buffer containing 50 mM Tris (pH 8.0), 5 mM CaCl_2_, and 0.02% NaN_3_. The gels were stained with 0.2% Coomassie blue R250 (GE Amersham, Piscataway, NJ, USA) and destained with 1:1 acetic acid/methanol solution. Experiments were performed in triplicate. Zymographic bands, which are indicative of MMP activity, were quantified by scanning densitometry.

### Statistical analyses

The normality of variable distributions was determined using the Shapiro-Wilk test. Associations between *MYC*, *FBXW7*, and *TP53* copy number variation, mRNA levels, protein expression, clinicopathological features, and cell invasion and migration capability were analyzed using the chi-square (χ^2^) and Mann–Whitney tests. Correlation between expression of the different target mRNAs was determined using Spearman’s test, in which a value below 0.3 indicated a weak correlation, 0.3-0.7 indicated a medium correlation, and values above 0.7 indicated a strong correlation. Data are shown as the median and interquartile range; p values less than 0.05 were considered significant.

## Results

### Gastric tumor specimens showed amplification of *MYC* and deletion of *FBXW7* and *TP53*

Three or more copies of *MYC* were found in 51.5% (17/33) of gastric tumor cells. In contrast, 45.5% (15/33) and 21.2% (7/33) of gastric tumor cells contained only one copy of *FBXW7* and *TP53*, respectively.

The association between clinicopathological features and *MYC*, *FBXW7*, and *TP53* copy number is summarized in Table 1. One gastric tumor that contained three copies of *TP53* was excluded from the chi-square analysis. No association was found between copy number variation of the genes studied and clinicopathological features.

### *MYC* mRNA expression was higher in tumors than in non-neoplastic specimens, whereas *FBXW7* and *TP53* mRNA expression was lower in tumor specimens

The expression level of *MYC* mRNA (2.01 ± 1.72 fold change) in tumor tissue samples was significantly higher than in non-neoplastic tissue (p = 0.0002), whereas the expression level of *FBXW7* mRNA (0.53 ± 0.40 fold change) and *TP53* mRNA (0.84 ± 0.55 fold change) in tumor tissue specimens was significantly lower than in non-neoplastic tissue (p < 0.0001 and p = 0.0011, respectively). We did not find a significant correlation between *MYC*, *FBXW7*, and *TP53* mRNA expression (*MYC*/*FBXW7* mRNA r = -0.3464, p = 0.0562; *MYC*/*TP53* mRNA r = 0.0950, p = 0.6113; *FBXW7*/*TP53* mRNA r = -0.0745, p = 0.4747). Thus, only a tendency toward correlation between an increase in *MYC* mRNA expression and a decrease in *FBXW7* mRNA expression was detected.

Table 2 summarizes the associations between various clinicopathological features and the RQ of *MYC*, *FBXW7*, and *TP53* mRNA expression in tumor and paired non-neoplastic specimens. An increase in *MYC* mRNA level was associated with the presence of lymph node metastasis (p = 0.016) and GC tumor stage III-IV (p = 0.036). A significant reduction in *FBXW7* mRNA level was also associated with the presence lymph node metastasis (p = 0.015) and tumor stage III-IV (p = 0.008).

**Table 2:** ***MYC, FBXW7 *and *TP53 *mRNA expression levels and clinicopathological factors of 33 gastric cancer patients**

	**n (%)**	***MYC***	***p- value***	***FBXW7***	***p- value***	***TP53***	***p- value***
		**Median ± IQR**		**Median ± IQR**		**Median ± IQR**	
**Age (y) (mean ± SD)**							
>50 (65.3 ± 9.1)	19 (57.6%)	2.04 ± 1.35	0.8873	0.55 ± 0.37	0.9247	0.86 ± 0.62	0.7409
≤50 (42.1 ± 8.2)	14 (42.4%)	1.44 ± 4.88		0.53 ± 0.50		0.94 ± 1.65	
**Gender**							
Male	15 (45.5%)	2.01 ± 1.01	0.4065	0.53 ± 0.22	0.6353	0.89 ± 0.58	0.8125
Female	18 (54.5%)	1.67 ± 2.03		0.56 ± 0.56		0.87 ± 0.69	
**Histopathology**							
Intestinal	22 (66.7%)	2.06 ± 0.99	0.3525	0.53 ± 0.16	0.1391	0.81 ±0.78	0.3311
Diffuse	11 (33.3%)	1.40 ± 2.32		0.77 ±0.74		0.94 ± 0.38	
**Depth of tumor invasion**							
T1	6 (18.2%)	0.89 ± 0.47	0.0857	0.88 ± 0.58	0.0678	0.85 ± 0.15	0.7069
T2-T4	27 (81.8%)	2.08 ± 1.42		0.53 ± 0.43		0.91 ± 0.79	
**Lymph node metastasis**							
Absent	13 (39.4%)	0.98 ± 1.09	0.0225*	0.68 ± 0.36	0.0238*	0.84 ± 0.44	0.6121
Present	20 (60.6%)	2.10 ± 2.20		0.46 ± 0.38		0.94 ± 0.79	
**Stage**							
I-II	18 (54.5%)	1.39 ± 1.23	0.0362*	0.57 ± 0.38	0.0380*	0.83 ± 0.55	0.0892^†^
III-IV	15 (45.5%)	2.41 ± 2.79		0.34 ± 0.45		0.96 ± 1.19	
**MYC IHC**							
Positive	20 (60.6%)	2.18 ± 1.59	0.0022*	0.53 ± 0.32	0.4090	0.81 ± 0.57	0.1372
Negative	13 (39.4%)	0.89 ± 0.85		0.58 ± 0.53		0.99 ± 0.90	
**p53 IHC**							
Positive	7 (21.2%)	4.25 ± 6.53	0.0891	0.64 ± 0.68	0.9203	1.06 ± 0.83	0.2937
Negative	26 (78.8%)	2.00 ± 1.44		0.55 ± 0.35		0.87 ± 0.60	

*p < 0.05; IQR: interquartile range.

### Nuclear MYC protein staining is associated with intestinal-type GC

**Figure 1:**
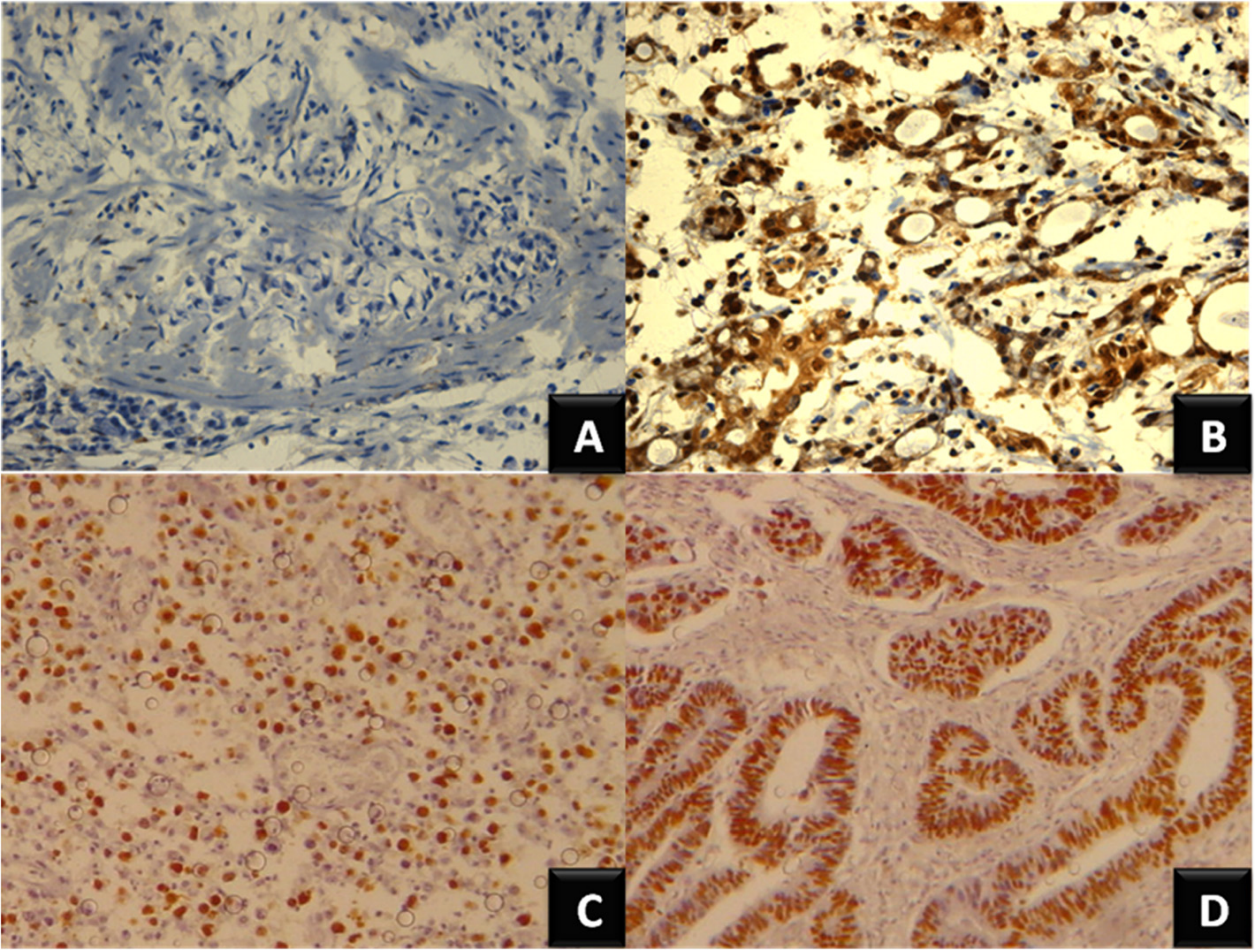
**Immunohistochemical analysis of MYC and p53 protein expression in GC. (A)** Negative MYC immunostaining in diffuse-type GC; **(B)** MYC immune positivity in intestinal-type GC; **(C)** Positive p53 immunostaining in diffuse-type GC; **(D)** p53 immune positivity in intestinal-type GC (magnification × 40).

Positive staining for nuclear MYC and p53 was found in 64.5% (20/31) and 19.4% (6/31) of GC samples, respectively (Figure 1). No positivity was found for FBXW7. Table 1 summarizes the clinicopathological features and MYC and p53 immunostaining results. Expression of MYC was more frequent in intestinal-type than diffuse-type GC (p = 0.007). Furthermore, MYC immunostaining was associated with increased *MYC* mRNA level (p = 0.0022). No association was found between p53 immunostaining and clinicopathological characteristics, *TP53* copy number, or *TP53* mRNA expression.

### Comparison of ACP02 and ACP03 cell lines

Both ACP02 and ACP03 cells contained three *MYC* copies and only one *FBXW7* copy. The number of *TP53* copies was undetermined in both cell lines. Compared with mRNA expression in ACP03 cells, ACP02 cells expressed a higher level of *MYC* (1.34-fold) and lower levels of *FBXW7* and *TP53* mRNA (0.62- and 0.73-fold, respectively).

**Figure 2:**
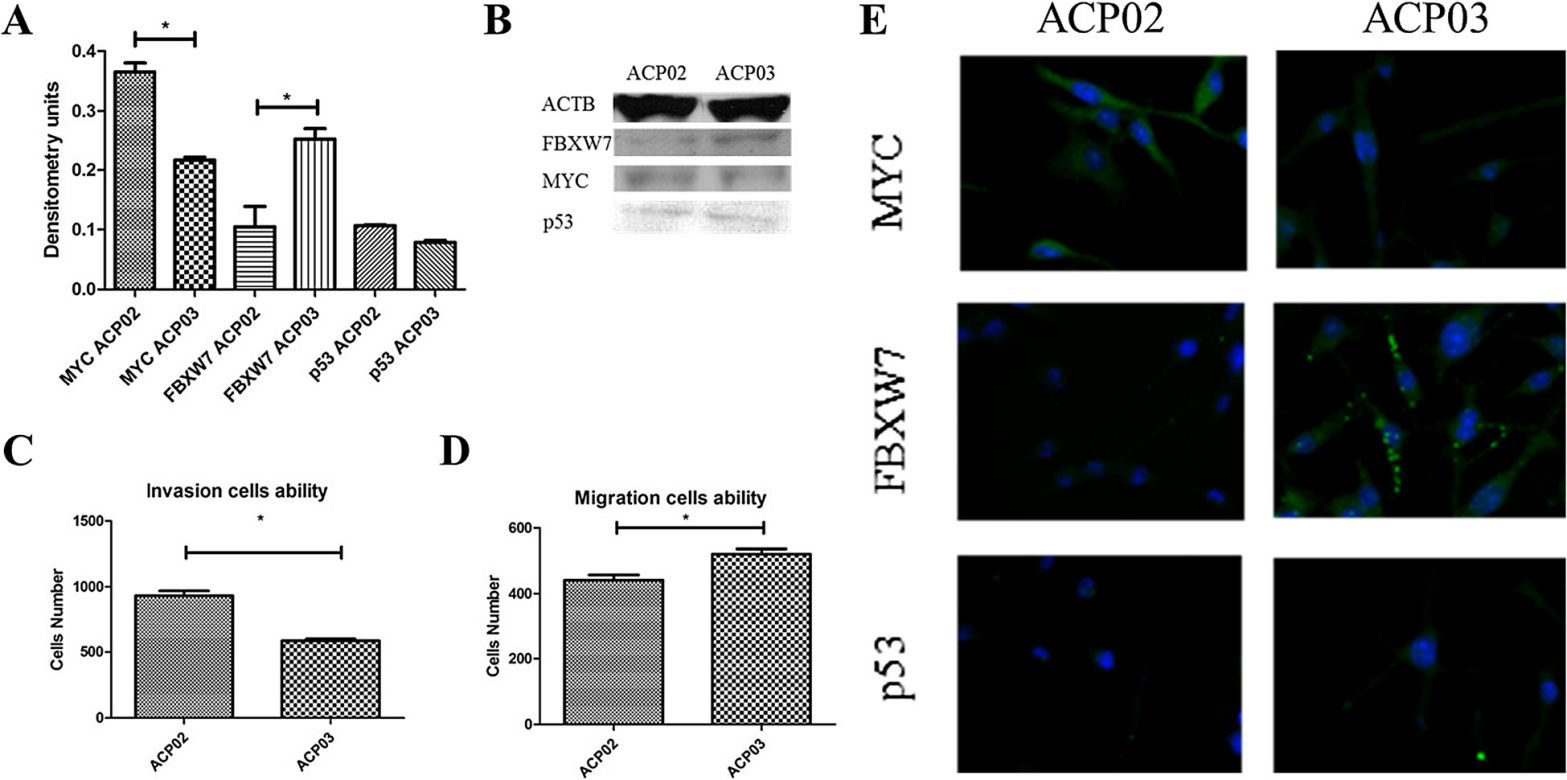
**MYC, FBXW7 and p53 expression, migration and invasion ability in ACP02 and ACP03. (A)** Graph show mean ± SD of MYC, FBXW7 and p53 protein expression in ACP02 and ACP03. These proteins were normalized to the level of beta actin; **(B)** Representative data of MYC, FBXW7 and p53 protein expression; **(C-D)** Graphs show mean ± SD of migration and invasive cells triplicates assay; **(E)** Representative results of MYC, FBXW7 and p53 immunofluorescence.

Western blot analyses revealed that MYC expression was significantly higher in ACP02 cells than ACP03 cells (p = 0.048). Moreover, FBXW7 expression was significantly lower in ACP02 cells than ACP03 cells (p = 0.049). However, there was no significant difference in p53 expression between the cell lines (p = 0.077) (Figure 2A-B).

Immunofluorescence analysis of both proteins showed a punctiform pattern of labeling, supporting the Western blot results showing an increase in MYC and reduction in FBXW7 expression in ACP02 cells compared with ACP03 (Figure 2). Matrigel invasion assay results showed that ACP02 cells were more invasive than ACP03 cells (p = 0.001). Migration assay results showed that fewer ACP02 cells migrated compared with ACP03 cells (p = 0.0028) (Figure 2C-D).

**Figure 3:**
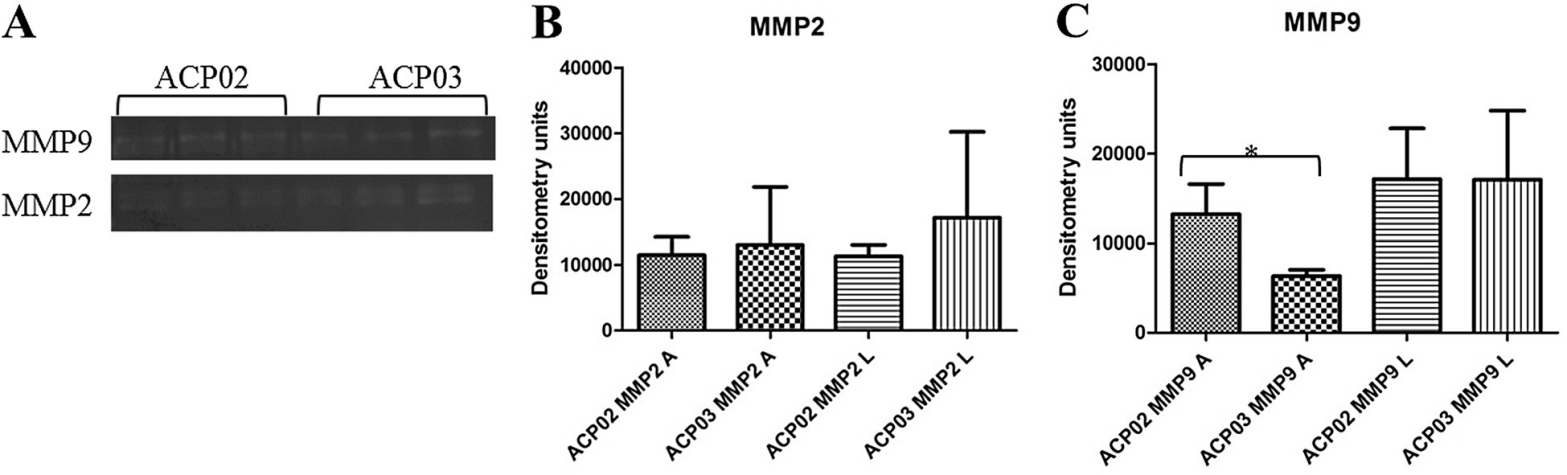
**Representative gelatin zymography analysis of MMP-2 and MMP-9 activity in ACP02 and ACP03. (A)** Bands corresponding to both latent and active forms of MMP-2 and MMP-9 were observed in ACP02 and ACP03. Densitometric analyses are of the bands corresponding to latent and active forms of MMP-2 **(B)** and MMP-9 **(C)**.

Both ACP02 and ACP03 cells presented four gelatinase activity bands: MMP-9 latent (92 kDa), MMP-9 active (88 kDa), MMP-2 latent (72 kDa), and MMP-2 active (66 kDa) (Figure 3). We found no significant differences in MMP-9 latent (p = 0.9788), MMP-2 active (p = 0.7848), and MMP-2 latent (p = 0.1678) between ACP02 and ACP03 cells. However, significant differences were found between ACP02 and ACP03 cells with respect to MMP-9 active (p = 0.0182).

## Discussion

In the current study, we observed that *MYC* mRNA expression was increased in GC samples compared with corresponding non-neoplastic samples. In addition, to our knowledge, this is the first study to report an association between increased *MYC* mRNA expression and the presence of lymph node metastasis and CG stage III-IV, reinforcing the idea that *MYC* deregulation is a strong factor for malignancy in GC.

Adams *et al*. [20] and Leder *et al.*[21] demonstrated that *MYC* mRNA expression deregulation can promote the development of cancer in transgenic mouse models. The increase in *MYC* mRNA level in human cancers may result from both direct and indirect mechanisms, which could have several explanations. First, *MYC* amplification is the most common mechanism of *MYC* deregulation in GC [5]. This mechanism leads to increased production of oncogenic products in quantities that exceed the transcriptional capacity of a normal double copy gene. Here, we observed three or more *MYC* gene copies in 51.5% of gastric tumors specimens. Previous studies from our group also showed that *MYC* amplification or trisomy of chromosome 8, on which *MYC* is located, was present in all GC samples examined from individuals in Northern Brazil, as well as in GC cell lines established by our group from tumors of Brazilian patients [18,22-27]. The presence of *MYC* amplification has also been reported in plasma samples from individuals with GC [28]. However, no direct association between *MYC* copy number variation and mRNA expression was detected in the present study.

Second, the increase in *MYC* mRNA expression may result from consistent recombination between the immunoglobulin (Ig) locus and the *MYC* oncogene. This phenomenon is frequently described in Burkitt’s lymphoma and is associated with a longer half-life of *MYC* mRNA in affected cells [29]. Previously, our research group observed *MYC* insertions in diffuse-type GC mainly into chromosomes that are mapped to genes of immunoglobulins (chromosomes 2, 14, and 22) [26]. Thus, chromosomal translocations involving the *MYC* locus (8q24) in diffuse-type CG in individuals from Northern Brazil might also reflect an increase in *MYC* mRNA level.

Immunohistochemistry (IHC) analysis revealed that MYC expression is more frequently found in intestinal-type GC than diffuse-type GC specimens. These alterations could lead to an abnormal MYC protein that is not recognized by either of the antibodies used in the present study. Moreover, we observed an association between *MYC* mRNA expression level and MYC staining. Furthermore, posttranscriptional mechanisms control MYC stability [6,30]. *MYC* deregulation has been associated with loss of *FBXW7*, a haploinsufficient tumor suppressor gene. In general, *FBXW7* loss may be caused by loss of heterozygosity (LOH) and mutation [30]. The loss at 4q, the *FBXW7* locus, is a recurring chromosomal alterations in GC [31,32], and *FBXW7* mutations have been found in 3.7-6% of gastric tumors [12].

In the present study, we observed only one copy of the *FBXW7* gene in 45.16% of the gastric tumors studied. Interestingly, *FBXW7* mRNA expression in GC samples is markedly decreased in comparison with corresponding non-neoplastic tissue. In addition, *FBXW7* mRNA expression deregulation was associated with the presence of lymph node metastasis and GC stage III-IV, as was also observed with *MYC* mRNA. These findings corroborate the work of Yokobori *el al.*[13], which also showed an association between reduced *FBXW7* mRNA expression and lymph node metastasis that contributes to the malignant potential of GC cells and results in poor prognosis. Moreover, we observed that the expression of *MYC* and *FBXW7* mRNA tended to be inversely correlated in the present study.

Several studies showed that *MYC* inactivation suppresses tumors in animals, suggesting that *MYC* may be a molecular target in cancer treatment [33-35]. Alternatively, Soucek *et al*. [36] proposed that FBXW7 might facilitate “tumor dormancy therapy”. Thus, MYC degradation by FBXW7 may not only induce a state of tumor dormancy but could also have an anti-tumor effect. Normally, MYC accumulation resulting from FBXW7 loss or another mechanism of MYC deregulation induces p53-dependent apoptosis via MDM2 degradation. The inactivation of both FBXW7 and p53 promotes MYC accumulation and inhibits p53-dependent apoptosis via MDM2 activation, which may in turn induce cell proliferation [37,38].

In this study, we found that 21.2% of the gastric tumors examined had one copy of the *TP53* gene and also found a substantial decrease in *TP53* mRNA level in GC tissues compared with paired non-neoplastic gastric tissue samples. Loss of p53 function could be caused primarily by LOH and mutations. *TP53* mutations in somatic cells are observed in about 50% of human cancers, but the frequency and type of mutation varies from one tumor to another and can be exchange of sense, nonsense, deletion, insertion, or splicing mutations [39,40]. In CG, the rate of mutations in this gene is 18-58% [41-43]. Some studies have shown that most missense mutations in *TP53* cause changes in the conformation of the protein, thereby prolonging its half-life and leading to accumulation in the nucleus of neoplastic cells. This accumulation can be detected by IHC in about 19-29% of GC tumors [44]. Here, we observed p53 immunostaining in 19.4% (6/31) of GC samples. This finding was consistent with earlier studies by our group that described LOH of *TP53* and deletion of 17p as frequent alterations in GC cell lines and primary gastric tumors from individuals in Northern Brazil [27,45]. The LOH may be related to the reduction of *TP53* mRNA expression observed in some of our GC samples. However, no association was found between this protein, *TP53* mRNA level, copy number, or clinicopathological features. The lack of association between *MYC*, *FBXW7*, and *TP53* copy number variation and mRNA and protein expression observed in this study highlights the complex relationship between gene copy number, mRNA expression, and protein stability.

In our previous cytogenetic study using fluorescence in situ hybridization (FISH), we described gains in *MYC* copies and deletions in *TP53* in ACP02 and ACP03 gastric adenocarcinoma cell lines, thus corroborating the present results obtained using real-time qPCR [27]. Both alterations were observed in the primary tumors from which these cell lines were established. Since ACP02 and ACP03 cells present alterations similar to those of gastric tumors, these cell lines may be useful as tools for experimental modeling of gastric carcinogenesis and may enhance understanding of the genetic basis underlying GC behavior and treatment and perhaps may change the landscape of GC.

In the present study, we also observed increased *MYC* and reduced *FBXW7* mRNA and protein expression in ACP02 cells compared with ACP03 cells. Furthermore, ACP02 cells were more invasive than ACP03 cells. On the other hand, ACP03 cells had a higher migration capability than ACP02 cells. Thus, despite the ability to migrate, ACP03 cells probably do not have efficient invasive machinery such as active proteases necessary to degrade the substrate. These findings are in agreement with observations in gastric tumors and reinforce the hypothesis that deregulation of MYC and FBXW7 is crucial for the invasive ability of GC cells. This result encouraged us to investigate the MMP-2 and MMP-9 activities of cells using zymography. The MMPs are synthesized as latent enzymes and later activated via proteolytic cleavage by themselves or other proteins in the intracellular space. Both proteases are synthesized predominantly by stromal cells rather than cancer cells and both contribute to cancer progression [46]. Our zymography analysis revealed no significant differences in the activity of MMP2 between ACP02 and ACP03 cells. Additionally, MMP-9 was more active in ACP02 than ACP03 cells. Studies have shown that high levels of MMP-2 and/or MMP-9 are significantly correlated with GC invasion and are associated with poor prognosis [47,48]. Sampieri *et al.*[49] showed that MMP-9 expression is enhanced in GC mucosa compared to non-neoplastic mucosa and that gelatinase activity differs significantly between cancerous and normal tissue.

## Conclusions

In conclusion, our findings show that *FBXW7* and *MYC* mRNA levels reflect the potential for aggressive biologic behavior of gastric tumors and may be used as indicators of poor prognosis in GC patients. Furthermore, MYC can be a potential biomarker for use in development of new targets for GC therapy.

## Competing interests

The authors declare that they have no competing interests.

## Authors’ contributions

DQC, VMF, ASK, MACS, AKRS and RRB participated in conception and design of study. DQC, VMF, SD participated in acquisition and performed the analysis of data. DQC, VMF, MFL, SD, CRTS performed interpretation of data. DQC, VMF, RM, PPA and RRB involved in drafting the manuscript. All authors read and approved the final manuscript.

## Pre-publication history

The pre-publication history for this paper can be accessed here:

http://www.biomedcentral.com/1471-230X/13/141/prepub
